# SMARCA4-Deficient Carcinoma of Uterine Cervix Resembling SCCOHT—Case Report

**DOI:** 10.3389/pore.2021.1610003

**Published:** 2021-12-14

**Authors:** Igor Sirák, Jan Laco, Hana Vošmiková, Loren K. Mell, Fernanda G. Herrera, Mária Šenkeříková, Milan Vošmik

**Affiliations:** ^1^ Department of Oncology and Radiotherapy, Faculty of Medicine and University Hospital, Charles University, Hradec Kralove, Czechia; ^2^ The Fingerland Department of Pathology, Faculty of Medicine and University Hospital, Charles University, Hradec Kralove, Czechia; ^3^ Department of Radiation Medicine and Applied Sciences, University of California, San Diego, La Jolla, CA, United States; ^4^ Ludwig Institute for Cancer Research, Lausanne University Hospital, University of Lausanne, Lausanne, Switzerland; ^5^ Department of Medical Genetics, Faculty of Medicine and University Hospital, Charles University, Hradec Kralove, Czechia

**Keywords:** cervical cancer, diagnostic biomarker, predictive marker, gynecological cancer, high-risk, personalized treatment, case report

## Abstract

Small cell carcinoma of hypercalcemic type (SCCOHT) is a rare gynaecological neoplasm, originating mostly in the ovaries. Cervical origin of this very aggressive malignancy with unknown histogenesis is an extremely rare condition, without published management recommendations. Alterations in *SMARCA4* gene are supposed to play the major role in SCCOHT oncogenesis and their identification is crucial for the diagnosis. Adequate genetic counselling of the patients and their families seems to be of great importance. Optimal management and treatment approaches are not known yet but may extremely influence the prognosis of young female patients that suffer from this very resistant disease. Nowadays, a translational research seems to be the key for the further diagnostic and treatment strategies of SCCOHT. The purpose of the case report is to provide practical information and useful recommendations on the diagnosis, management, and treatment of SMARCA4-deficient carcinoma of the uterine cervix resembling SCCOHT.

## Introduction

Small cell carcinoma of the ovary (SCCO) is a very lethal malignancy. It contains two separate subtypes: the pulmonary type (SCCOPT), and hypercalcemic type (SCCOHT) [1].

SCCOHT is one of the most frequent malignant undifferentiated ovarian tumors in women younger than 40 years. It is a rare and highly lethal tumor that typically affects young women. Many women are diagnosed at early stage. However, the aggressive behaviour of SCCOHT leads to a significantly bad prognosis, while most women die within 1 year after the diagnosis is established. The prognosis is bad even for very early stage SCCOHT.

SCCOHT was first described in 1982 in a series of 11 patients with concurrent SCCO and hypercalcemia [2]. Several hundred cases have been described in the English literature to date. In the largest published series, 99% tumors were unilateral with a higher frequency in the right ovary (66%); average age of diagnosis is 24 years (range 9–43 years), and most patients also present with hypercalcemia (62%) [3].

Familial SCCOHT cases may be more often bilateral [4,5]. Hypercalcemia may be associated with paraneoplastic production of parathyroid hormone or parathyroid hormone-related peptides [6,7]. In a smaller number of published cases, patients had significant symptoms of hypercalcemia including severe pancreatitis or altered mental status [8,9]. Paraneoplastic hypercalcemia usually normalizes after surgical removal of the tumor. Even without laboratory or clinical signs of hypercalcemia, the term “hypercalcemic type” has been applied to this tumor type to distinguish it from ovarian SCCOPT, a high-grade neuroendocrine tumor.

A NCDB study (National Cancer DataBase) showed a high frequency of elevated CA125 (84% of cases) [10]. Serum CA125 levels in SCCOHT may not elevate as clearly as in ovarian epithelial carcinomas. Extraovarian dissemination and lymph node metastases are seen in approximately half of the cases [3].

SCCOHT is well described in the miscellaneous ovarian tumors chapter in the recent WHO Classification of Female Genital Tumours (2020). Grossly, the tumor is usually unilateral, large, solid, and tan to white to grey. Foci of haemorrhage, cystic degeneration, and necrosis are common. Microscopically, the tumor is composed of monomorphic cells, arranged mostly in solid and, less commonly in follicular, trabecular, or nested growth pattern. The follicle-like spaces may contain eosinophilic or basophilic secretions. In most cases, the predominant tumor population consists of cells with round, ovoid, or occasionally spindled hyperchromatic nuclei with coarsely clumped chromatin and small nucleoli and with scant cytoplasm (so-called “small blue round cell” appearance). Mitotic activity is typically brisk and proliferation index Ki67 is regularly high. In about half of the tumors, larger cells with eccentric vesicular nuclei with prominent nucleoli and with abundant eosinophilic cytoplasm (so-called rhabdoid appearance) are present. If significantly predominant, a “large cell variant” of the tumor may be considered. In up to 15% cases, a minor mucinous component either in the form of benign glands or cysts, or rarely as malignant signet-ring cells is present. The stroma of the tumor is usually minimal, it may be, however, myxoid or edematous in occasional cases [11]. Immunohistochemical profile of the tumor is relatively nonspecific[12].

Recently, loss of SMARCA4 (a.k.a. BRG-1) expression, due to germline or somatic mutation of *SMARCA4* gene, was reported in more than 90–95% of cases and is currently considered an important diagnostic finding. However, not all tumors are negative as occasional neoplasms show loss of both SMARCB1 and SMARCA2 with retained SMARCA4 expression. Dual loss of SMARCA4 and SMARCA2 is not uncommon in these tumors, the latter occurring due to epigenetic inactivation rather than mutation [12].

Differential diagnosis depends on the proper localization of the tumor and includes, in principle, any poorly differentiated malignant tumor. Regarding malignancies of the uterine cervix, particularly poorly differentiated adenocarcinoma, adenosquamous or squamous cell carcinoma, malignant melanoma, small cell neuroendocrine carcinoma, SMARCA4-deficient undifferentiated uterine sarcoma, dedifferentiated endometrial carcinoma, non-Hodgkin malignant lymphomas, high-grade leiomyosarcoma and rhabdomyosarcoma, Ewing sarcoma, and germ cell tumors (e.g., embryonal carcinoma and yolk sac tumor) must be considered. As regards more common ovarian localization, particularly the juvenile variant of granulosa cell tumor needs to be distinguished [11], as well as a malignant teratoid or rhabdoid tumor of the ovary [13,14].

As regards ovarian carcinoma, e.g., *BRCA1/2* germline or somatic mutations play the major role in development of high-grade serous adenocarcinoma, the most common malignancy of the ovary [15]. However, SCCOHT has not been reported to harbour *BRCA1/2* mutations. Similarly, classic oncogene mutations (e.g., *KRAS*/*BRAF*) have not been identified in SCCOHT yet. On the other hand, SCCOHT is distinctively characterized by *SMARCA4* mutations (germline or somatic), or mutations in other subunits of the SWI/SNF complex [16,17]. The majority of SCCOHT cases were prooved to be driven by inactivating mutations of *SMARCA4* gene, that is involved in the SWI/SNF complex [16–18].

SCCOHT has typically a low mutation load with low PD-L1 (Programmed Death Ligand-1) expression, which makes this cancer less suitable for immune checkpoint blockade treatment. However, the response of SCCOHT to modern immunotherapy has to be studied, with some positive response already reported [19].

Next generation sequencing (NGS) technologies as Whole Exome Sequencing (WES) and Whole Genome Sequencing (WGS) can distinguish driver and passenger mutations or non-pathogenic normal gene polymorphisms in ovarian carcinomas [20].

More than a hundred of pathogenic *SMARCA4* mutations have been studied to reveal the presence of different types of gene alterations [11]. These *SMARCA4* specific mutations consisted of frameshift (36.4%), stop/nonsense (32.2%), splice-site (20.3%), missense (5.9%) and in-frame deletions (5.1%), with no concrete mutational hotspot defined.

In all mentioned studies, immunohistochemical SMARCA4 (a.k.a. BRG1) protein expression was lost in 82–95% of SMARCA4 altered tumors, confirming inactivating mutations of SMARCA4 gene with defective protein production. For comparison, only 0.4% of other primary ovarian tumors have negative SMARCA4 protein expression.

Other SCCOHT tumors with intact SMARCA4 protein expression may carry mutations in other SWI/SNF complex subunits, e.g., inactivating SMARCB1 (alias INI1), or ARID1A mutations [21]. However, SCCOHT is mostly associated with SMARCA4 and seldom with SMARCB1 mutations, or others.

To maintain the chromatin remodeling activity of the SWI/SNF complex in human, three core subunits (INI1/SMARCB1, BAF155/SMARCC1, and BAF170/SMARCC2) and two helicases/ATPases (BRM/SMARCA2 and BRG1/SMARCA4) are essential [22–25]. To control chromatin accessibility, the SWI/SNF complex binds to DNA regions via ARID1A and ARID1B.

The mammalian SWI/SNF complex functions as a tumor suppressor, and the subunits of the mammalian complex (encoded by *ARID1A, PBRM1, SMARCB1, SMARCA4*, and *ARID2* genes) are frequently mutated in human cancers. SWI/SNF complex was found to be mutated in circa 20% of human malignancies according to a meta-analysis [26]. Protein SMARCA4 is the most frequently mutated chromatin remodeling ATPase in cancer.

### Treatment of Ovarian SCCOHT

Surgical resection of the primary tumor with expert gynecologic pathology review is widely recommended by recent management guidelines [12]. The operable patients should be treated with surgery including total abdominal hysterectomy and bilateral salpingo-oophorectomy. Omentectomy, lymph node dissection, debulking of extra-uterine disease, and peritoneal biopsies may be advocated in some of the patients. Fertility-conserving surgery is not feasible because of the aggressive behaviour of the tumor. However, some may advocate that the poor estimated survival may justify a less radical procedure without worsening the outcome. The average overall survival was 35 months in patients at stage I, and only 3 months at stage IV [27].

Patients older than 40 years have a worse survival than younger ones, but there is no significant outcome difference between patients with and without germline *SMARCA4* mutations [28]. Most of the tumors recur rapidly and shortly after surgery with a typical clinical course including pulmonary metastases and respiratory distress. Although, some treatment response is typically seen after four cycles of palliative chemotherapy, but massive recurrence is noted shortly thereafter.

No prospective randomized phase III studies have been conducted to date in SCCOHT. With dose-intensive chemotherapy approach, a 3-year survival rate of 49% was reported among SCCOHT patients [29].

Adjuvant systemic treatment of SCCOHT is not standardized and achieves only a modest improvement of survival. The standard chemotherapy for ovarian carcinoma (paclitaxel/carboplatin or BEP–bleomycin/etoposide/cisplatin) is applied in most SCCOHT patients, with no clearly proven benefit [3]. However, a German study demonstrated that 4/7 patients achieved a complete response for 7–73 months with the treatment of conventional chemotherapy [30]. Adjuvant combined chemotherapy with vinblastine/cisplatin/cyclophosphamide/bleomycin/doxorubicin/etoposide (VPCBAE) was also published, with no clear benefit or recommendation [31]. Some authors suggest chemotherapy similar to the treatment of soft-tissue sarcoma, e.g., gemcitabine plus docetaxel, or doxorubicin plus ifosfamide. There is also some published experience with irinotecan [32], cisplatin and cyclophosphamide and etoposide; or topotecan and platinum and paclitaxel [33]. However, there is no widely accepted consensus on the standard adjuvant nor palliative treatment for this lethal cancer, based on monocentric experience. The International SCCOHT consortium recommends BEP or VPCBAE cytotoxic chemotherapy or high-dose chemotherapy as the first choice in newly diagnosed disease; and cyclophosphamide/doxorubicin/vincristine, or carboplatin/paclitaxel, or topotecan in the recurrent disease [12].

On the other hand, some *in vitro* studies showed SCCOHT cell lines quite resistant to platinum chemotherapeutic drugs. On the contrary, epothilone demonstrated a strong anti-proliferation effect *in vitro* and in xenografts *in vivo* [34,35].

Multi-modality intensive treatment approaches combining surgery, high-dose multiagent chemotherapy (with eventual stem cell transplantation), and postoperative radiotherapy may be an adequate treatment option for most SCCOHT patients [36,37]. Survival after surgery may be better with postoperative high-dose chemotherapy and stem cell rescue, compared with conventional chemotherapy alone [28]. Patients with additional radiotherapy achieved a better survival than patients with surgery and chemotherapy alone. Therefore, radical surgery with postoperative multiagent chemotherapy and radiotherapy may be the best treatment option for most patients, sometimes associated with a good prognosis.

Several agents such as bortezomib, pazopanib, or PARP inhibitors were described as possible treatment options for SCCOHT [38]. Immunotherapy with PD-1/PD-L1 checkpoint blockage is a standard of care in many immunogenically sensitive cancer types with high tumor mutation load and high PD-L1 expression. Unfortunately, SCCOHT is not a typical hypermutated cancer, nor does it have high PD-L1 expression [21,34]. On the other hand, the immunogenic microenvironment and published experience may provide some rationale for checkpoint immunotherapy, as several SCCOHT patients with tumor PD-L1 expression have responded well to anti PD-L1 therapy [19]. However, long-lasting treatment response to pembrolizumab in SMARCA4 deficient tumor with negative PD-L1 expression has also been reported [39]. Among SWI/SNF deficient tumors, ARID1A deficiency may contribute to impaired mismatch repair, leading to increased tumor mutational burden and increased response to immune checkpoint blockage [40,41].

SCCOHT displays notable genomic stability and does not seem to acquire additional mutations after exposure to chemotherapy [42]. Combined loss of SMARCA4 and SMARCA2 in SCCOHT cell lines may induce an extreme sensitivity to EZH2 inhibitors *in vitro* and *in vivo* [43–47]. At the same time, SCCOHT cells are reported to be highly sensitive to the inhibition of cyclin-dependent kinase 4/6 (CDK4/6) *in vitro*, therefore CDK4/6 inhibitors should be also studied in this indication [48].

The available literature about radiotherapy in SCCOHT consists of smaller case series and single case reports. Some series suggest that postoperative radiotherapy may play an important role in the primary treatment of SCCOHT [3,33]. Additional reports also support the role of radiotherapy in the treatment of recurrent SCCOHT [49]. To date, there has been no case of successful definitive radiotherapy alone in SCCOHT treatment published. Overall, the evidence for standard use of radiotherapy in SCCOHT treatment is lacking due to the rarity of the disease.

### Case Description

**FIGURE 1:**
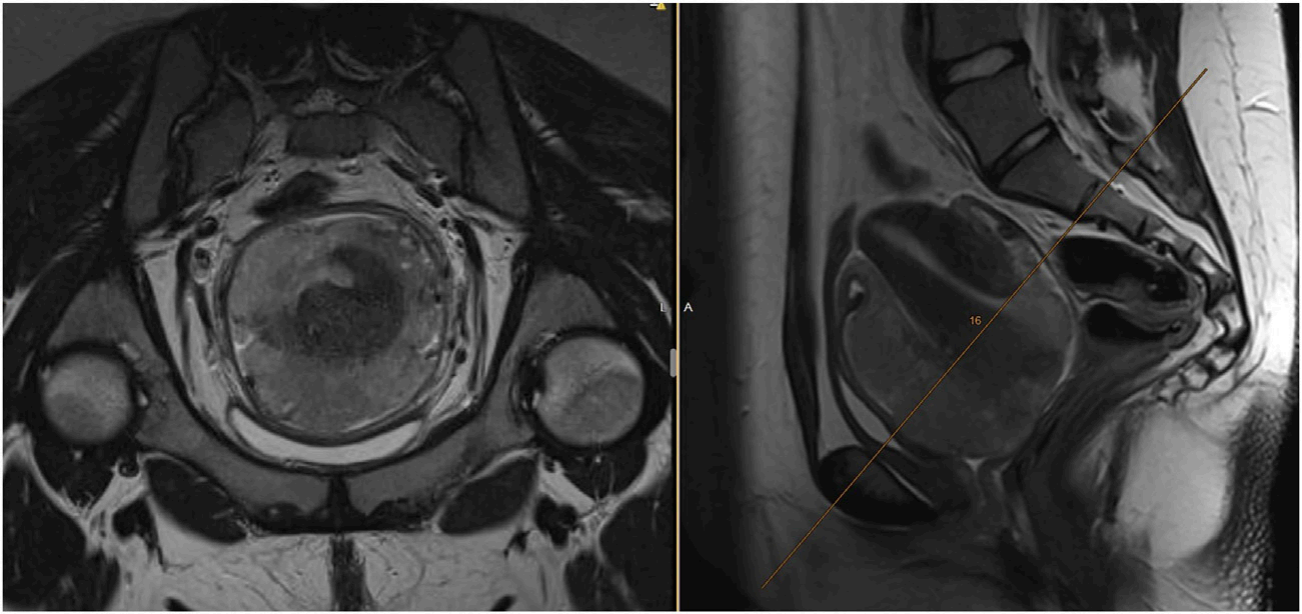
Baseline MRI of the uterine cervix SCCOHT. The cervical tumor grows atypically more around the uterus, than infiltrating it. Expansive tumor growth with pressing to the bladder or rectum, with no clear infiltration into adjacent organs.

An 18-year-old female, virgo intacta, came to a gynaecologist for metrorrhagia. During the examination, a tumor of the cervix was found. Immediate biopsy was performed, and the patient was referred to the onco-gynaecological centre. Magnetic resonance imaging (MRI) of the pelvis revealed a tumor measuring 80 × 90 × 80 mm, growing from the cervical region, *de facto* resembling the uterus (Figure 1), filling the vagina, pressing on the bladder and rectum, without clear invasion into adjacent organs. At the same time, presacral lymphadenopathy (30 × 15 × 15 mm) was present, as well as bilateral iliac lymphadenopathy (28 × 21 × 40 mm). A very rare, small cell carcinoma, hypercalcemic type (large cell subtype) was confirmed by biopsy. The baseline level of serum calcium was normal.

During explorative laparotomy, the removal of lymphadenopathy with histologically confirmed metastases of hypercalcemic small cell carcinoma was successful. However, radical hysterectomy without residual disease was impossible, unless the patient underwent anterior pelvic exenteration. The patient was immediately indicated for chemotherapy via our multidisciplinary team. In the absence of any treatment recommendations, first-line cisplatin plus etoposide combined chemotherapy was selected, with regular pelvic MRI before each subsequent cycle to monitor the treatment response. After 2 cycles of chemotherapy, progression of the disease in the uterus was found, with infiltration of bilateral parametria, the bladder wall and anterior rectal wall infiltration. Distant dissemination on whole-body CT was not clearly demonstrated, except for non-specific small lesions of both lungs up to 5 mm.

**FIGURE 2:**
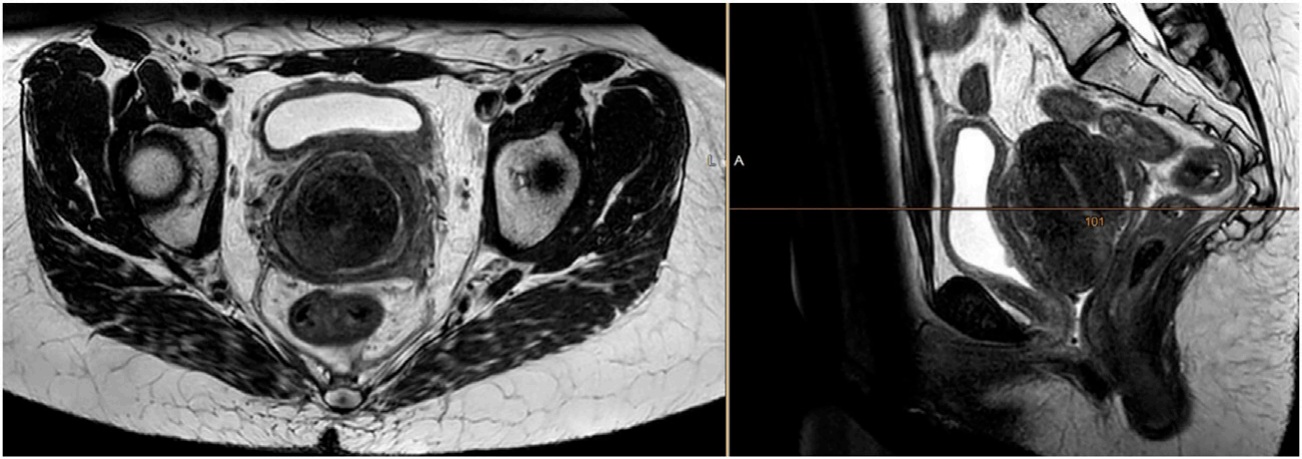
MRI confirming a partial tumor response 1 month after completion of radiotherapy at a dose of 59.4 Gy in 33 fractions. The treatment response was obvious, however incomplete.

Due to gynaecological bleeding and micturition problems, the patient underwent extended-field pelvic radiotherapy with retroperitoneal lymph-nodes up to 45 Gy in 25 fractions, with a subsequent boost to the tumor, uterus and vagina up to a total dose of 59.4 Gy in 33 fractions. The pattern of tumor growth outside the uterus was not suitable for intracavitary brachytherapy. The patient managed radiotherapy without complications with clinical relief from both bleeding and urinary symptoms. The partial regression of both the tumor and lymphadenopathy was confirmed by MRI (Figure 2).

**FIGURE 3:**
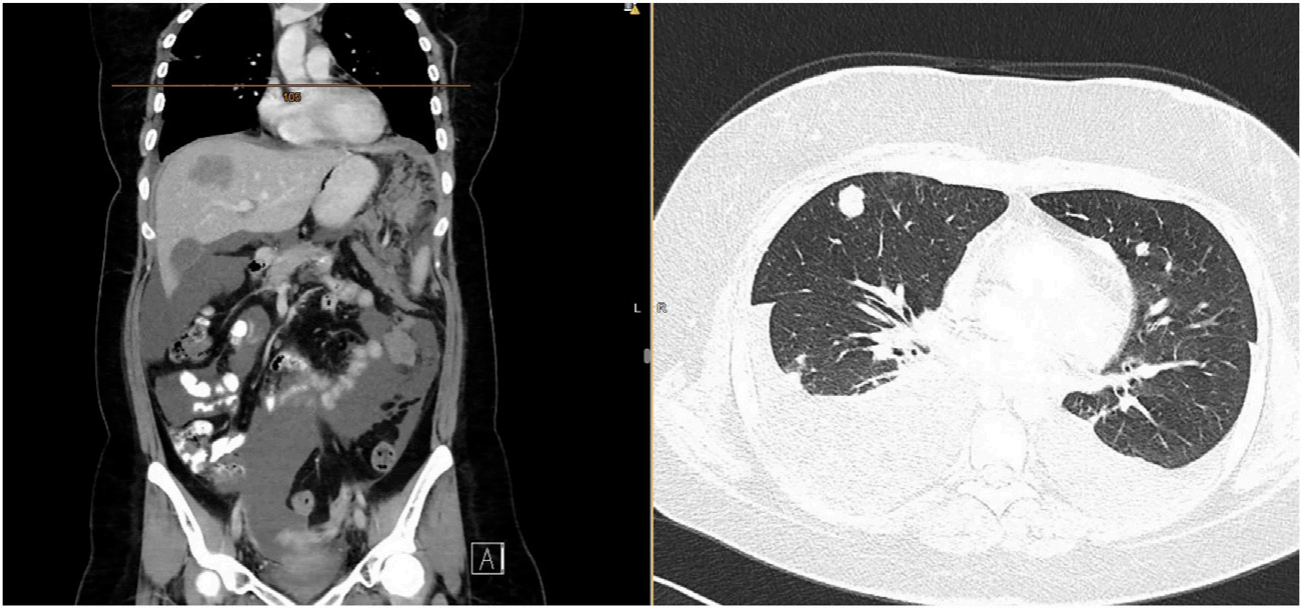
CT scan confirming rapid tumor dissemination with omental, liver, lung, and pleural metastases, with ascites and bilateral pleural effusion.

Three months after radiotherapy, 7 months from the initial diagnosis, the patient was admitted to the hospital for general deterioration, breathlessness, fever, loss of appetite, and pain. Immediate CT of the head ruled out brain metastases. Although partial regression of the pelvic tumor was confirmed on the whole-body CT scan, there was massive systemic progression of the disease with mediastinal and neck lymphadenopathy, multiple metastatic lesions of the lungs and pleura with bilateral pleural effusions, multiple liver metastases (largest 52 × 36 mm), omental involvement with soft tissue masses (largest 72 × 56 mm), and ascites (Figure 3). The patient died due to an extremely rapid disease progression before 2nd line palliative chemotherapy could start.

In the present case, the diagnosis of hypercalcemic small cell carcinoma (large cell subtype was based on the microscopic appearance of the tumor in hematoxylin and eosin staining and the result of immunohistochemical examination).

**FIGURE 4:**
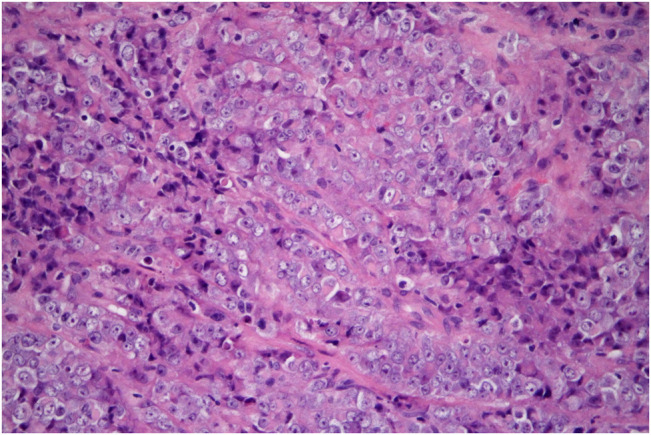
SMARCA4-deficient carcinoma of uterine cervix resembling SCCOHT. The tumor consists of cells with enlarged vesicular nuclei with clearly visible nucleoli and abundant eosinophilic cytoplasm. It is so-called large cell, or rhabdoid, variant of this tumor (hematoxylin-eosin, original magnification 400x).

Microscopically, the tumor was composed entirely of large cells with vesicular, less commonly hyperchromatic nuclei, frequently with prominent nucleoli, and with abundant eosinophilic cytoplasm, sometimes imparting a rhabdoid appearance (Figure 4). Small cell component, characteristic for “classic” variant of SCCOHT, was absent. Follicle-like spaces or mucinous components were not observed. Microscopic appearance of lymph node metastases was identical to the tumor seen in the cervical biopsy.

**FIGURE 5:**
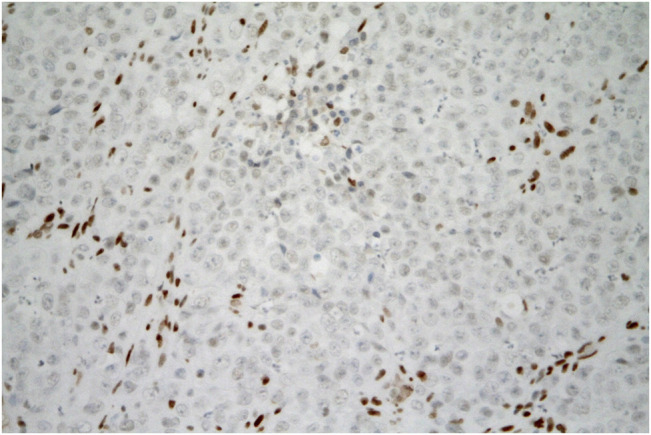
SMARCA4-deficient carcinoma of uterine cervix resembling SCCOHT. Tumor cells are negative for SMARCA4 expression. The brown staining of the nuclei of non-tumor fibroblasts and inflammatory cells can serve as a positive internal control. (immunohistochemistry, original magnification 400x).

Immunohistochemically, the tumor cells showed diffuse or nearly diffuse expression of broad-spectrum cytokeratins (CK), CK18, PTEN, β-catenin (membranous), vimentin, and GATA3. Expression of MLH1, PMS2, MSH2, and MSH6 was retained in tumor cell nuclei, indicating the absence of microsatellite instability (MSI). There was focal expression of p16. Isolated tumor cells showed expression of PAX8, CD10, calretinin, and synaptophysin. Detection of CK5/6, CK7, CK19, CK20, EMA, p63, p40, ER, PR, CEA, chromogranin, CD56, WT1, SALL4, Oct3/4, CD30, glypican-3, inhibin, SOX10, melan A, HMB-45, and LCA gave negative results. The expression of PD-L1 was negative in the tumor. The most diagnostically beneficial finding was the complete loss of SMARCA4 (a.k.a. BRG-1) expression (Figure 5). Expression of both ARID1A and SMARCB1 (a.k.a. INI-1) was maintained.

HPV DNA detection and genotypization was performed by qualitative real-time PCR with the AmoyDx Human Papillomavirus Genotyping Detection Kit (Amoy Diagnostics, China). In a DNA extract of the tumor tissue, no HPV DNA was detected.

#### Whole Exome Sequencing

Both germline and somatic WES of the patient and the tumor was performed. Library for whole exome capture and sequencing was prepared from DNA extracted from patient´s blood and tumor using TruSeq Exome Kit according to the manufacturer´s instructions. Prepared library was loaded onto NextSeq 500/550 Mid Output Kit v2.5 (150 cycles) and sequenced on the NextSeq 500 instrument (all Illumina, CA, United States). Sequencing coverage for exomes was >20× at > 90% of captured regions.

The mutation burden of the tumor was low (4 mutations/Mb). Examination of both the germline and somatic exome revealed the following selected variants (Table 1):

**TABLE 1:** Germline and somatic gene variants found by the Whole Exome Sequencing (WES).

Variants detected by germline WES				
Gene	Variant c.DNA/protein	Zygosity	gnomAD variant frequency	ACMG variant classification
*SMARCA4*	c.3976G>T/p.E1326*	Heterozygous	-	Likely Pathogenic (class 4)
*SMARCA4*	c.4199C>G/p.T1400R	Heterozygous	-	Uncertain Significance (class 3)
*PALB2*	c.509_510del/p.R170fs	Heterozygous	0.007%	Pathogenic (class 5)
*BRCA2*	c.8350C>T/p.R2784W	Heterozygous	0.0008%	Pathogenic (class 5)
*PRF1*	c.272C>T/p.A91V	Heterozygous	4.62%	Uncertain Significance (class 3); a risk allele
**Variants detected by somatic WES**				
**Gene**	**Variant c.DNA/protein**	**VAF**		**ACMG variant classification**
*FGFR3*	c.586C>T/p.R196C	7%		Uncertain Significance (class 3)

gnomAD variant frequency–Non-Finnish European population.

VAF, Variant Allele Frequency (frequency of the variant in the tumor sample).

ACMG classification, The American College of Medical Genetics and Genomics classification.

There were no other variants of the genes encoding SWI/SNF complex found, including *ARID1A* (wild-type), *SMARCA2* (wild-type), *SMARCB1* (common variables in non-coding sequencies), and others.

#### Family Genetic Counselling

As several germline likely pathogenic or pathogenic gene variants were detected by WES (*PALB2, BRCA2, SMARCA4*), genetic testing was recommended also to the patient’s family.

A heterozygous c.3976G>T/p.E1326* variant in the *SMARCA4* gene and c.8350C>T/p.R2784W variant in the *BRCA2* gene was detected in the mother of a patient (39 years old, no malignancy). Mother’s familial history was also negative for malignancy.

The patient’s father (42 years, no malignancy) showed a heterozygous c.509_510delGA/p.R170fs variant in the *PALB2* gene. Father’s father (a heavy smoker) died of lung cancer at the age of 51; the father’s mother died of an unknown malignancy at the age of 64 years; and the father’s brother died of pancreatic cancer at the age of 38 years.

The patient’s brother (23 years old, no malignancy) showed a familial heterozygous *PALB2* variant inherited from the father.

## Discussion and Recommendations

Here we report the very first published case of SMARCA4-deficient carcinoma of the uterine cervix resembling SCCOHT and our challenging diagnostic process and dismal treatment experience. The histological picture of the disease, as well as the severity of its behavior and insufficient response to treatment does not seem to differ from the experience we have with the ovarian type of the disease. It was an aggressive tumor that affected a very young woman, with an advanced stage at diagnosis, with a rapid progression and a typical severe course of the disease, with early dissemination to the liver, peritoneum, and lungs, which resulted in death within 8 months from the first symptoms.

The main uncertainity of this case study is whether the uterine cervix was the place of origin of such a rare histology. Uterine cervix is rarely the site of metastatic spread of other malignancies, but this cannot be excluded. On the other hand, vaginal metastases constitute the majority of vaginal malignancies, which mainly originate from the cervix, endometrium, or ovary, among other locations. Since the status of ovary/ovaries has never been examined histologically in our patient, we cannot exclude primary ovarian SCCOHT with cervical manifestation. There was no tumor mass present in the patient on ultrasound, CT, or MR imaging. Theoretically, the ovarian lesion can be subtle and may not be picked up by imaging. However, this possibility seems highly unlikely.

The patient was a virgin at diagnosis, excluding HPV associated tumorigenesis. Moreover, the detection of HPV DNA was negative in tumor tissue. Clinical manifestation was otherwise not atypical, instead of the primary tumor growing more around the uterus, than infiltrating it. It is crucial to distinguish SCCOHT of the uterine cervix from the much more common squamous cell carcinomas or adenocarcinomas, that is possible only after careful histopathological examination. The most diagnostic is the loss of SMARCA4 (rarely SMARCB2 or ARID1A) expression immunohistochemically.

However, the microscopic differential diagnosis is very broad and includes any poorly differentiated/high-grade malignant tumor of both the uterine cervix and the uterine corpus. Absence of HPV DNA excludes the diagnosis of both HPV-associated squamous cell carcinoma and HPV-associated adenocarcinoma. Moreover, markers of squamous differentiation, i.e., CK5/6, p63, and p40, were completely negative and the absence of CK7 and CEA expression would be unusual in cervical HPV-associated adenocarcinoma. Morphology of the tumor excludes the diagnosis of any of HPV-independent cervical adenocarcinomas, i.e., of gastric type, of clear cell type, and of mesonephric type. Large cell neuroendocrine carcinoma shows significant expression of CD56, chromogranin, and synaptophysin. In the present tumor, the expression of CD56 and chromogranin was completely negative, and synaptophysin was detected only in isolated tumor cells, making the diagnosis of large cell neuroendocrine carcinoma unlikely. Germ cell tumors should be also considered. However, the expression of SALL4 as well as of other markers (Oct3/4, CD30, glypican-3) was completely negative, arguing against this diagnosis. Absence of expression of LCA and of “melanoma” markers (SOX10, melan A, HMB-45) excludes the diagnosis of hematologic malignancy and malignant melanoma, respectively. As regards the uterine corpus, particularly dedifferentiated/undifferentiated endometrial carcinoma and recently described SMARCA4-deficient uterine sarcoma enter the differential diagnosis [50–53]. Dedifferentiated/undifferentiated endometrial carcinoma may be composed of large rhabdoid cells and may show the absence of expression of SWI/SNF complex components, including SMARCA4, in a significant number of cases. Another typical findings include the presence of microsatellite instability and only focal expression of cytokeratins (CK). In the present tumor, however, we observed nearly diffuse expression of CK and microsatellite instability was not found. In addition, we did not see the absence of PTEN expression or nuclear expression of β-catenin, which would indirectly indicate a mutation in the corresponding genes, which are common in dedifferentiated/undifferentiated endometrial carcinoma. Differential diagnosis of SMARCA4-deficient uterine sarcoma is very challenging as this tumor may be morphologically almost indistinguishible from hypercalcemic small cell carcinoma (large cell subtype). However, in the present case we found nearly diffuse expression of CK at CK18, which would be very unusual for SMARCA4-deficient uterine sarcoma as it is typically negative for these markers.

The overall mutation load of the tumor was typically low, as expected in SCCOHT. The most probable pathogenic variant associated with SCCOHT that was found in the patient was only the germline *SMARCA4* c.3976G>T/p.E1326* variant in a heterozygous form. This variant was inherited from the mother. It was present in the tumor, also in a heterozygous form. Other germline polymorphisms detected in the *SMARCA4* gene were also heterozygous in the tumor. For this reason, the loss of heterozygosity in the tumor, e.g., due to deletion of the second unmutated *SMARCA4* alelle, cannot be confirmed only considering WES data. However, a complete loss of SMARCA4 protein expression was confirmed immunohistochemically. Therefore, another processes that led to the loss of function of the second unmutated allele of the *SMARCA4* gene cannot be excluded (e.g., epigenetics).

The patient inherited a heterozygous mutation in the *SMARCA4* and *BRCA2* genes from her mother and at the same time a heterozygous mutation in the *PALB2* gene from her father. This very unlikely mutational load with an autosomal dominant inheritance might probably cause, by its nature, such a rare malignancy at such a young age. SCCOHT is described as a monogenic disease, the most probable cause of an extremely rare histological type of the tumor in the presented case, was the inherited likely pathogenic *SMARCA4* variant, with the loss of SMARCA4 protein function and expression. However, overall DNA repair instability, caused by *BRCA2* and *PALB2* variants could play a role in much more difficult “game of genes.”

Germline variants in *SMARCA4* imply familial SCCOHT with a requirement of genetic counselling, that should be recommended for all family members. However, there is no clinical management consensus on *SMARCA4* mutation carriers. Patients carrying germline mutations are usually younger (usually <50 years old) than noncarriers. In patients younger than 18 years old, the absence of germline mutation is unlikely. Considering the rapidly progressive tumor growth and high mortality of SCCOHT, germline mutation carriers should be counselled about prophylactic bilateral oophorectomy. However, the early onset of SCCOHT (usually before the first pregnancy) indicates that prophylactic surgery should be performed at a younger age than in hereditary *BRCA 1/2* variant carriers. Therefore, prophylactic bilateral oophorectomy may not be acceptable for young SMARCA4 variant carriers. An improvement on *in vitro* fertilization may facilitate prophylactic surgery in these carriers. Cervical/uterine SCCOHT is such a rare condition that prophylactic hysterectomy in young *SMARCA4* variant carriers, in our opinion, would not be justified. Our recommendation is to consult *SMARCA4* variant carriers about the risks considerately and omit hysterectomy in patients contemplating *in vitro* fertilization. For *in vitro* fertilization, pre-implantation genetic diagnosis should be recommended to prevent genetic transmission of the deleterious mutations.

Effective treatment options for SCCOHT are lacking. The rarity of SCCOHT limits the design of randomized clinical trials. Available evidence suggests the benefit of multi-modality treatment with radical surgery, high-dose multiagent chemotherapy with eventual stem cell transplantation. Postoperative radiotherapy may be advocated, considering retrospective case series and otherwise high rates of tumor recurrence and progression. However, clear evidence for the standard use of adjuvant irradiation is lacking and when used, we advise for the careful implementation of IMRT techniques sparing as much as healthy tissue as possible. Palliative directed radiotherapy may provide some treatment response with no proven curative potential. In our patient, a dose of 59.4 Gy in 33 fractions lead to a partial, incomplete response. Therefore, we strictly do not recommend definitive radiotherapy of SMARCA4-deficient carcinoma of the uterine cervix resembling SCCOHT with curative potential, as otherwise standardized in the treatment of advanced squamous cell carcinoma or adenocarcinoma.

Overall response of SCCOHT to standard chemotherapy regimens is poor, with rapid progression of the disease during or shortly after chemotherapy. In our patient, there was an early progression of the disease during platinum-based chemotherapy. Combined cisplatin/etoposide chemotherapy is a standard of care in lung small cell carcinoma. However, due to our experience, we do not recommend combined cisplatin plus etoposide as an effective treatment of cervical SCCOHT.

## Data Availability

The original contributions presented in the study are included in the article/supplementary material, further inquiries can be directed to the corresponding author.
